# Reciprocal Inhibitions of HRP and REC in a Coculture System

**DOI:** 10.22259/2638-5120.0601005

**Published:** 2025-08-07

**Authors:** Andrew Tsin, Laura Valdez, Richard LeBaron

**Affiliations:** 1 Neuroscience, The University of Texas Rio Grande Valley School of Medicine, Edinburg, Texas, United States.; 2 Neuroscience, Developmental and Regenerative Biology, The University of Texas at San Antonio, San Antonio, Texas, United States.

**Keywords:** Coculture, Diabetic Retinopathy, Reciprocal Inhibitions

## Abstract

One of the earliest hallmarks of diabetic retinopathy (DR) is the loss of retinal pericytes. Human retinal pericytes (HRP) are contractile cells that share a common basement membrane with retinal endothelial cells (REC). HRP envelop the REC to maintain tubular integrity and control the hydrostatic pressure of microcirculation. The loss of HRP leads to the development of advanced-stage DR pathology including angiogenesis. However, the mechanism of mutual cell support or inhibition remains unclear. In the present study, HRP and REC were evaluated for cell growth in a coculture system. Results from a monoculture system show that HRP had a shorter doubling time (DT) than REC, based on their growth curves (DT: 34 hr for HRP, 56 hr for REC). When HRP were cocultured with REC, DT of HRP increased from 34 hr (in the monoculture system) to 45 hr (in a coculture system). Similarly, when REC were cocultured with HRP, the DT of REC also increased from 56 hr to 77 hr. These results provide strong evidence for reciprocal inhibitions between HRP and REC in a coculture system. As HRP dropout occurs at the early stage of DR, the elimination of HRP inhibition on REC may lead to REC proliferation, ahead of the action of VEGF from retinal ischemia. Additional experiments that investigate the effects of the interactions of cocultured HRP and REC are expected to uncover details for further insight into the role of REC and HRP on DR.

## 1. Introduction

Diabetes is a highly prevalent health disparity disease characterized by hyperglycemia (1). Prolonged diabetes induces significant damage to the kidney, nervous system, heart and many other organs/systems. In the eye, chronic hyperglycemia leads to the development of diabetic retinopathy (DR), a leading cause of blindness in the world. There are approximately 93 million people who have been diagnosed in 2010 with DR worldwide (2). In the United States there are more than 37 million people with diabetes, and it was estimated there were around 10 million which had DR in 2021 (3). It is projected that the number of people with diabetes in the United States will increase to around 61 million by 2060.

As this number increases so will the associated complications such as DR (3). Prolonged diabetes, poor glycemic control and high blood pressure are strongly associated with DR (2, 4). Moreover, DR development in individuals with type 2 diabetes is more prevalent in Mexican Americans, with an estimate of 34%, compared to non-Hispanic white Americans (26%) (4). Previous studies show that DR not only affects the eye but also affects a person’s quality of life (5).

One of the earliest hallmarks of DR is the loss of retinal pericytes through apoptosis. Human retinal pericytes (HRP) are contractile cells that share a common basement membrane with retinal endothelial cells (REC) and provide support for REC. HRP are most prominent in retinal capillaries in comparison with capillaries in other tissues in the body (6–8). HRP envelope REC to aid in maintaining tubular integrity and control the hydrostatic pressure of the microcirculation. Early stages of DR development include a significant loss of HRP which leads to weakening of the integrity of the retinal capillaries and leakage of exudate. Furthermore, retinal ischemia leads to the increase of VEGF and endothelial cell proliferation and neovascularization.

Although there have been extensive studies on the interaction between HRP and REC, the underlying mechanism by which interactions between HRP and REC lead to cell proliferation or cell survival remain unclear. Only a limited amount of literature is available to describe how HRP/REC interact in a coculture system (9–15). Furthermore, the hypothesis that HRP can affect (enhance or inhibit) the growth of REC (or vice versa) has not been investigated in a coculture system. In the present study, growth curves from HRP and REC in a coculture system were obtained and compared to those in a single cell type (monoculture) system. Data from our HRP/REC coculture system revealed a strong, reciprocal inhibition of cell proliferation in comparison to mono (single cell type) culture.

## 2. Materials and Methods

### 2.1 Materials

Primary human retinal pericytes (HRP) (passage 3) and primary human retinal microvascular endothelial cells (REC) (passage 3) were purchased from Cell Systems (ACBR1830 and ACBR181, respectively). Complete Classical Medium (CCM) with serum and culture boost was purchased from Thermo Fisher (50-900-036). Transwell multiple well plates with permeable polyester membrane (PET) inserts were purchased from Corning (3782). A Countess 3 automated cell counter (Invitrogen) quantified the number of viable cells in trypan blue treated populations.

### 2.2 Methods

**Figure 1.**
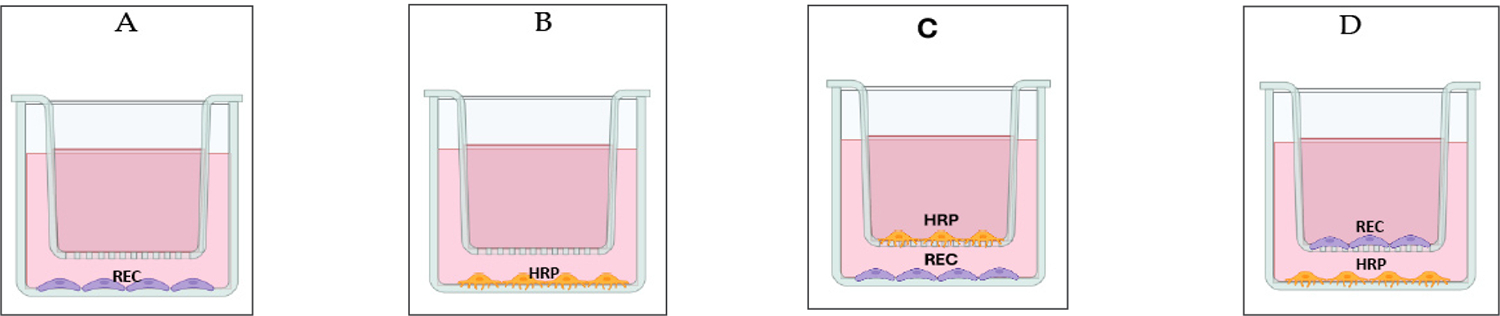
Schematics of HRP/REC Coculture System. HRP and REC from cell passages 5–8 were cultured using Complete Classic Medium (CCM) containing serum and CultureBoost^™^. For mono-cell culture, wells of a 24-well plate with transwell insert were used to culture on the lower chamber (the well surface) 60,000 REC (A), or in parallel, 60,000 HRP (B). For two-cell type, non-contact coculture system, cells were grown on transwell inserts holding 40,000 cells on a PET membrane in the upper chamber, and 60,000 cells grown on the well surface as indicated (C&D). Cell culture media in both chambers were changed at 24-hr intervals.

For routine maintenance and experiments, HRP and REC were incubated in a 37°C humified incubator with an atmosphere of ambient air and 5% CO_2_. In wells of a 24-well plate a transwell insert holding 40,000 cells grown on a PET membrane in the upper chamber (0.5 mL Complete Cell Media) and 60,000 cells grown onto the well (referred to as the lower chamber, 1 ml CCM), fashioned a two-cell type (non-contact) coculture system of HRP and REC (Fig 1 C&D). In contrast, a one-cell type mono-culture system holding 60,000 cells (either HRP or REC) were grown in a 24-well plate with a transwell insert and PET membrane only (Fig 1 A&B). Cell culture media in both chambers were changed every 24 hr. The number of viable cells were determined using a trypan blue dye exclusion method (% viability: 70–94) and provided the number of HRP and REC viable cells for monoculture and co-culture experiments. Duplicate data points were gathered at 0, 24, 48 and 72 hrs. Doubling time was calculated from the log phase of the growth curves. This experiment was repeated twice with similar results.

## 3. Results

### 3.1 Coculture System

Schematics of the HRP/REC mono and coculture systems are shown in Fig 1. Fig 1A shows REC monoculture, while Fig 1B illustrates HRP monoculture. Fig 1C shows that HRP cells were grown onto the transwell PET membrane with CCM separating HRP from REC that were grown onto the base of the well in a no-contact coculture model. Fig 1D shows that REC were grown onto the transwell PET membrane with CCM separating REC from HRP grown onto the base of the well in a no-contact coculture model.

### 3.2 Inhibition of HRP Growth in the HRP/REC Coculture System

**Figure 2.**
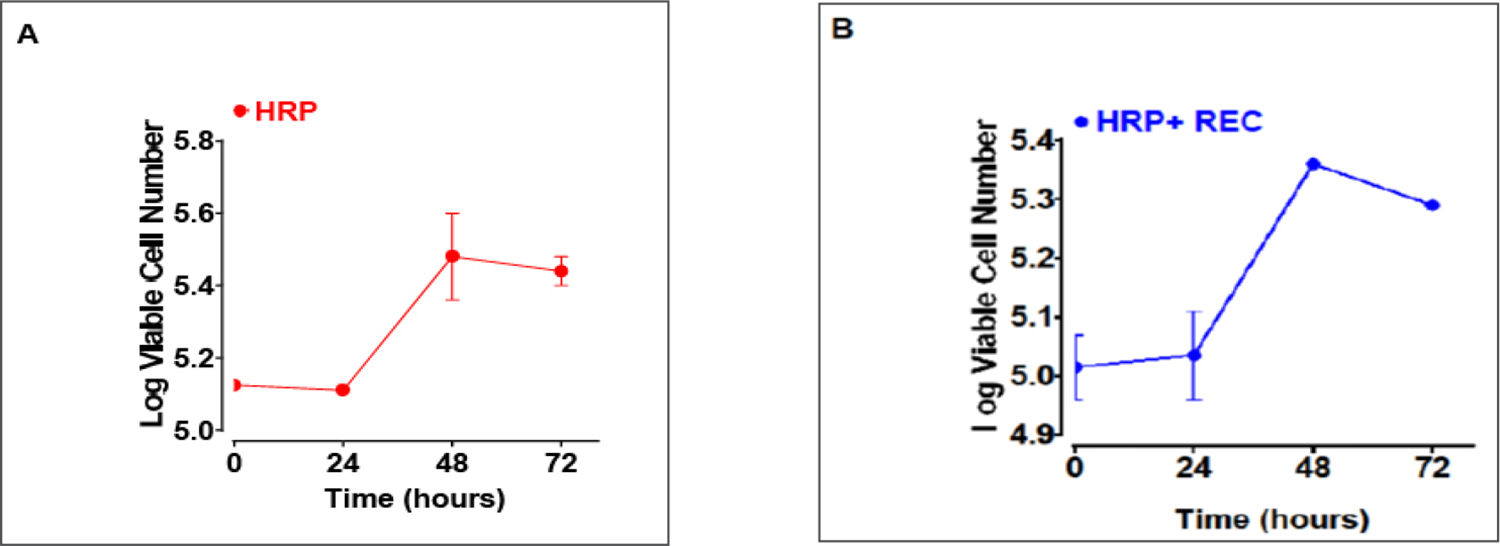
Inhibition HRP growth in the HRP/REC coculture system. HRP cells were seeded on the lower chamber of a 24-well plate at a seeding density of 60,000 cells/1ml without REC (one-cell type culture system, see Fig 1 B) and with 40,000 REC (two-cell type coculture system, see Fig 1D). Cells were incubated 24 hr and the number of cells determined (designated as time zero). Media was changed every 24 hr and viability was determined via trypan blue exclusion assay. The number of viable HRP increased from 60,000 to 134,500 within 24 hr (from seeding to time zero) in the monoculture system (A). In contrast, the number of viable HRP increased from 60,000 to 104,500 within 24 hr (from seeding to time zero) in the two-cell type coculture system (B). The range of two determinations in the number of viable HRP cells (in both mono vs two-cell culture systems) at 0, 24, 48 and 72 hr time points are indicated in the figure. HRP doubling time was 34 hr when it was grown without REC (A), whereas when HRP was cocultured with REC, the doubling time increased to 45 hr. The vertical axis in this figure is plotted in log scale and the horizontal axis in linear scale.

In the monoculture system the number of viable HRP cells was 134,500 at time zero, 129,000 at 24 hr, 313,500 at 48 hr, and 275,000 at 72 hr (Fig 2A). During the log phase (from 24 to 48 hr), the doubling time was determined to be 34 hr (Fig 2A). In the coculture system, the number of viable HRP cells was 104,000 at time zero, 110,500 at 24 hr, 229,000 at 48 hr and 197,000 at 72 hr (Fig 2B). The doubling time was calculated to be 45 hr at the log phase (Fig 2B). The increase in HRP doubling time in the coculture system suggests an inhibition of HRP proliferation from their interaction with REC.

### 3.3 Inhibition of REC Growth in the HRP/REC Coculture System

**Figure 3.**
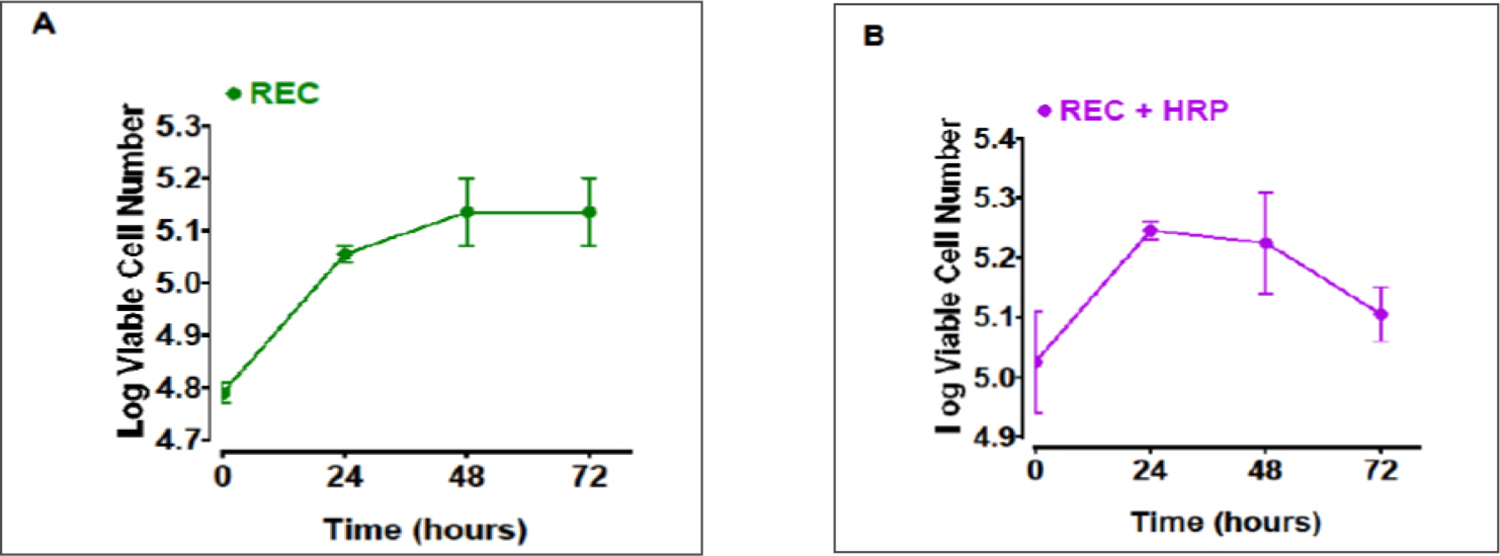
Inhibition of REC growth in the HRP/REC coculture system. REC cells were seeded on the lower chamber of a 24-well plate at a seeding density of 60,000 cells/1ml without HRP (one cell type culture system, see Fig 1A) and with 40,000 HRP cells (two cell type coculture system, see Fig 1C). Cells were incubated 24 hr and the number of cells determined (designated as time zero). Media was changed every 24 hr and viability was determined via trypan blue exclusion assay. Viable REC number increased from 60,000 to 61,300 within 24 hr (from seeding to time zero) in the mono cell type system (A), whereas viable REC number increased from 60,000 to 107,600 within 24 hr (from seeding to time zero) in the two-cell type coculture system (B). The range of two determinations in the number of viable REC cells (in both mono vs two-cell culture systems) at 0, 24, 48 and 72 hr time points are indicated in the figure. REC doubling time was 56 hr when grown without HRP (A). However, when REC were cocultured with HRP, the doubling time increased to 77 hr (B). The vertical axis in this figure is plotted in log scale and the horizontal axis, in linear scale.

In the monoculture system, the number of viable REC cells was 61,300 at time zero, 113,500 at 24 hr, 138,500 at 48 hr and 138,500 at 72 hr (Fig 3A). During the log phase (from 0 to 24 hr), the doubling time was derived to be 56 hr (Fig 3). In the coculture system, the number of viable REC cells was 107,600 at time zero, 175,000 at 24 hr, 172,500 at 48 hr and 129,500 at 72 hr (Fig 3B). The doubling time was calculated to be 77 hr at the log phase (Fig 3). The increase in REC doubling time in the coculture system suggests an inhibition of REC proliferation from their interaction with HRP.

### 3.4 Increase in the Doubling Times in the HRP/REC in the Coculture System

**Figure 4.**
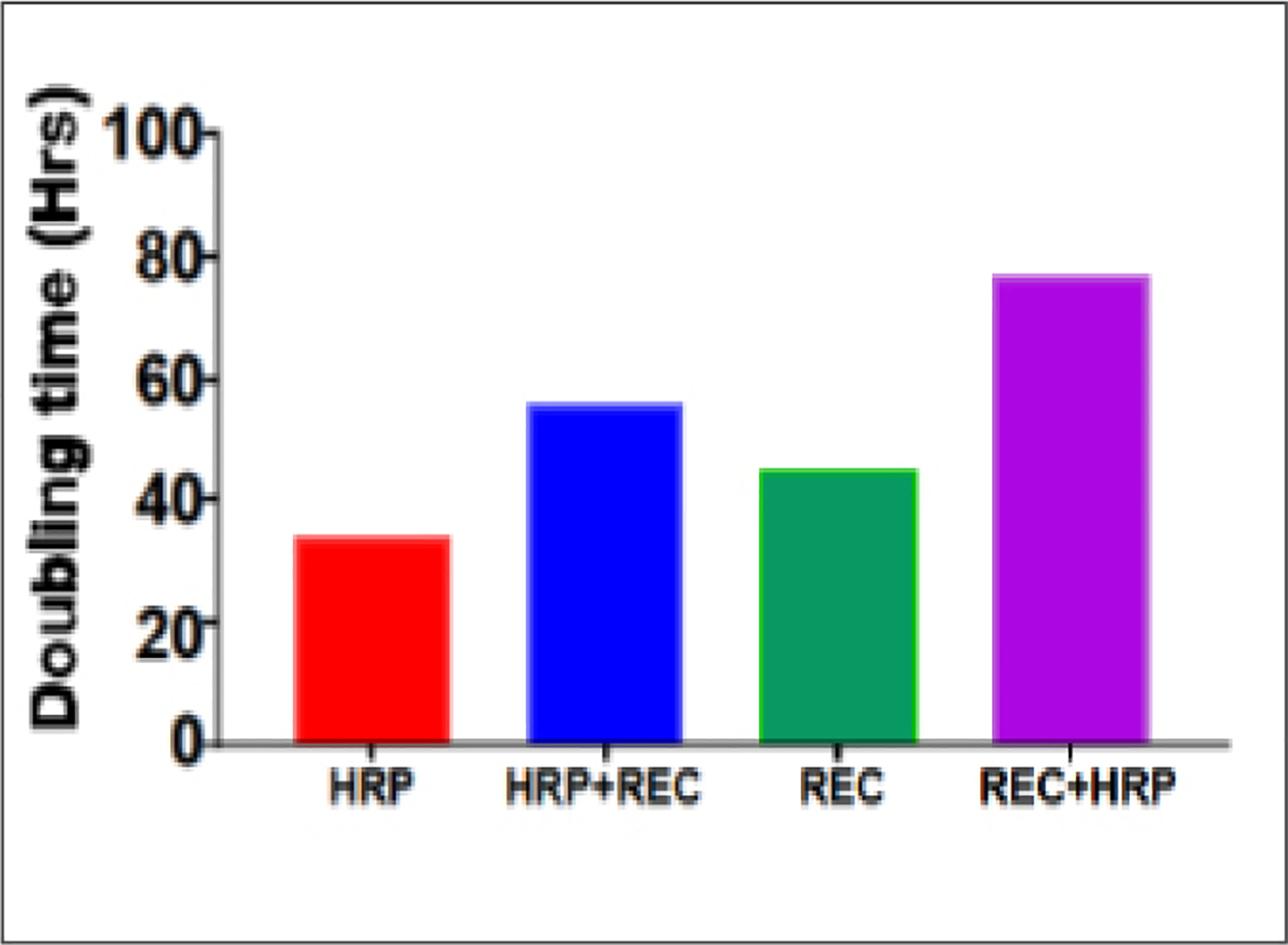
Increase in the doubling times in the HRP/REC in coculture system (from monoculture). Doubling time for monoculture of HRP and REC were derived from growth curves as described in Fig. 2 and 3. Doubling time for coculture of these two cell types were derived from growth curves of each cell type when they were grown together in a non-contact system. In wells of 24-well plates, a transwell insert apparatus holding 60,000 REC in the lower chamber and 40,000 HRP on an upper chamber fashioned a two-cell type, no-contact, coculture system. Additionally, a transwell insert apparatus holding 60,000 HRP in the lower chamber and 40,000 REC on the upper chamber fashioned a coculture system. Cells were incubated as described in methods. For experiments, after a 24-hr incubation period the time course (time zero) began. Based on a large increase in the REC doubling time in the two-cell type coculture system (from 56 to 77 hr), the presence of HRP had significantly inhibited the growth rate of REC. In comparison, there was also an increase in the HRP doubling time in the two-cell type coculture system (from 34 for mono to 45 hr for two-cell systems), thus the presence of REC had also significantly inhibited the growth rate of HRP.

Figure 4 shows the progression of cell growth and proliferation (based on doubling time from the growth curves) between HRP and REC in monoculture versus coculture. In mono (single cell type) cultures, HRP exhibited a faster growth rate than REC, based on a shorter doubling time of 34 hr (HRP), versus 48 hr (REC). In comparison to monoculture, the growth rate of HRP decreased when they were cocultured with REC (doubling time increased from 34 to 56). Likewise, REC growth rate also decreased when they were cocultured with HRP, as evident by a large increase (45 hr to 77 hr) in doubling time.

## 4. Discussion

Pericytes play a key role in regulating blood-barrier permeability and the absence of pericytes in pathological conditions such as DR coincides with increased permeability Because pericytes surround the endothelium and act as a physical barrier that contributes to a well-developed blood retinal barrier, pericyte apoptosis leaves the endothelium prone to increased permeability and injury (7). The retina has the highest pericyte density of all vascular beds (6). Studies show that high glucose decreases human retinal pericyte (HRP) viability (7) and increases apoptosis in bovine retinal pericytes (16, 17). In humans, the HRP-to-REC ratio decreases from 1:1 in healthy retinas to 1:0.5 in diabetic retinas (7, 18), which suggests that HRP act as one of the first lines of defense. Loss of HRP is one of the earliest morphological changes in DR (19), and it is also associated with an increase in ghost cells, shunt vessels and microaneurysms (7).

Although cultured HRP and REC have been used to study their role on the development of diabetic retinopathy, only a few studies are available to show how these cell types interact in a coculture environment (9–12, 15). Moreover, there has not been a detailed report on the growth rate/proliferation of these cells in coculture. In our present study, we constructed the complete growth curve of HRP and REC in the absence and presence of the other cell type by monoculture and coculture systems. HRP and REC grown in monoculture had a larger increase in cell numbers within the 72 hr incubation period and exhibited a faster rate of cell growth (lower doubling time) than those grown in the coculture system with the other cell type. Thus, we conclude that when cocultured, HRP and REC reciprocally inhibit each other in cell growth and proliferation. We are particularly interested in the inhibition of REC growth by HRP, because HRP are known to drop out at the early stage of DR. From this data we form a novel hypothesis in which HRP dropout removes HRP-mediated inhibition on REC growth, thereby adding to the effect of VEGF-stimulation of REC growth, leading to neovascularization and angiogenesis (i.e. proliferative DR).

Based on recent reports on cross talks between HRP and REC (20–24), it is possible that HRP inhibits REC growth by action of exosomes to mitigate the overall or a specific metabolic function in REC. Although several exosomes have been identified in recent reports on the upregulation of certain exosomes in diabetic conditions (22, 24) the exact identities of exosomes from HRP, and their actions on REC remain unknown. Furthermore, our coculture system will provide an excellent model to test our hypothesis, and further our investigations on the role of a hyperglycemic environment, exosomes, ECM molecules, and cytokines (TGFs), on HRP apoptosis, in the presence and absence of REC. Such examinations on the effect of cell-to-cell inhibition/interactions at the molecular level in our HRP/REC coculture system will lead to new information to better understand the effect of HRP apoptosis on REC proliferation in the development of DR.
